# Recycling some byproducts for fabrication of green cement with good mechanical strength and high efficiency for wastewater treatment

**DOI:** 10.1038/s41598-024-79009-7

**Published:** 2024-11-13

**Authors:** Fatma M. Helmy, S.M.A. El-Gamal, M. Ramadan, F. A. Selim

**Affiliations:** Chemistry Department, Faculty of Science, Ain Shams University, Cairo, Egypt

**Keywords:** Blast furnace slag, Silica fume, Alkali-activated cement, Mechanical characteristics, Dyes removal, Hydrothermal treatment, Energy science and technology, Engineering, Materials science

## Abstract

This research aims to produce green cement, as an alternative to traditional cement, with outstanding performance. Five alkali-activated cement pastes were fabricated based on NaOH-activation of slag (GGBFS), bypass (B), and/or silica fume (S). Codes of five pastes are C, C-20B, C-30B, C-10B10S, and C-20B10S, as C is the control paste containing 100% slag. The compressive strength of the fabricated pastes was measured at different curing regimes: Conventional curing for 3 months and autoclave curing at 4 bar/153◦C, 7 bar/178◦C, and 10 bar/198◦C for 4 h. XRD, TGA/DTG, SEM/EDX, and BET/BJH techniques were utilized to clarify the phase development, morphological and texture features of the formed alkali-activated composite pastes. Besides, the removal capacity of some pastes for methylene blue and indigo-carmine dyes from aqueous media was evaluated. The results confirmed that C and C10B10S (80%GGBFS + 10%B + 10%S) pastes have significant mechanical properties and distinctive meso-porosity that can remove both anionic and cationic dyes.

## Introduction

Portland cement manufacturing is an extensive energy-consuming and high greenhouse gas emitting product. Cement industries release about 7% of their carbon dioxide gas into the atmosphere, or 50% of all industrial emissions of carbon dioxide gas^1^. Besides, various toxic gases, including Sulphur dioxide, nitrogen oxide, and dust, are released throughout the cement-making process^2,3^. Dust produced from the cement industry is released into the atmosphere, contributing to environmental degradation. Cement dust severely impacts human health, the ecosystem, and vegetation. To address these concerns, the building industry must discover a partial or full replacement for OPC as an alternative to cementitious materials^4^. Additionally, rapid industrial growth results in a loss of natural resources, induces greenhouse gas emissions and pollution. By utilizing additional cementitious materials, such as metakaolin (MK), fly ash (FA), and silica fume (SF), the problems associated with such depletions and global warming can be resolved^5^. Geopolymers or alkali-activated cement, are fabricated by alkali-activating natural minerals or solid wastes that are aluminosilicate-rich, like ground granulated blast furnace slag^6^, fly ash^7^, silica fume^8^, bypass^9^, red mud^10^, and metakaolin^11^. The production of alkali-activated materials (AAMs), in contrast to Portland cement, does not require a calcination process, which drastically lowers the used energy, limits CO_2_ emissions, and permits the use of a wide range of industrial solid wastes in addition to a plentiful supply of inexpensive raw materials^12^. Moreover, AAMs have demonstrated more endurance than Portland cement under harsh conditions, including fire^7^, sewage^13^, and chemical acid^14^. As a result of the superior performance and less environmental effect of geopolymers and/or alkali-activated materials (AAM) compared to regular Portland cement (OPC) concrete, they can be considered a prospective substitute.

There is a fundamental difference between geopolymers and AAMs. Geopolymers are generated from the alkali-activation of alumino-silicate materials of low CaO content. Materials include fly ash, silica fume, metakaolin, bentonite, glass wastes, red mud, rice husk ash, and red brick waste. The alkali-activation of these materials generates sodium alumino silicate hydrates (NASH) as the main hydraulic phase^15–17^. On the other hand, AAMs are produced from the alkali-activation of alumino-silicate materials rich in high percentages of CaO, such as slag. The alkali-activation of slag produces calcium silicate hydrate (CSH), calcium alumino silicate hydrate (CASH) and hydrotalcite-like phase; these phases are responsible for quick strength development, quick setting and good durability^18,19^.

Cement bypass flue dust (CBFD) is a by-product of cement manufacture and is created in rotatory kilns during clinker preparation. CBFD is rich in alkaline oxides and chlorides that may increase the alkalinity of OPC, so it cannot be recycled with raw material feed^20^. Silica fume is a byproduct solid created during the smelting of silicon metal and the production of ferro-silicon alloy. Ferro-silicon alloy is made up of a large amount of fine and amorphous silicon dioxide (SiO_2_) elements. Silica fume is the most widely used cementitious material. It is made up of tiny particles with surface areas ranging from 13,000 to 30,000 m^2^/kg^21^. Aydın et al. investigated the effects of ternary combinations of GGBFS/FA/SF-based alkali-activated cements on water sorptivity, flexural strength, and compressive strength. The findings demonstrated that a ternary mixture including 20% FA, 19% SF, and 61% GGBFS generated the best cementing performance^22^. Collins and Sanjayan studied the impact of ultrafine silica fume on the workability and strength development of alkali-activated slag cement (AASC). It was found that replacing slag with 10%SF enhanced the compressive strength after one day, while the flowability was lower compared to the control^23^. The mechanical properties and microstructure of AASC made with silica fume activator (SFA) have also recently drawn the attention of numerous researchers. SFA was combined with an alkaline solution, and the generated solution was more active than a solution of NaOH or Na_2_Si_2_O_3_^24–26^. It has been discovered that the production of high-quality CSHs, in addition to a marked densification in the pore structures of slag/fly ash-based alkali-activated cement were attributed to the utilization of SFA as an alkaline activator which was a successful alternative to sodium silicate^27^.

Many studies have talked about cement kiln dust because it contains a high percentage of calcium oxide, so it is recycled again into raw materials in the cement industry^28^. On the other hand, not much research has been published on recycling cement bypass to produce blended cement due to its high alkalinity. Few studies have addressed its use in the fabrication of alkali-activated cement. Abiad et al.^29^ confirmed that bypass dust can be used as an alkaline activator. Mixing slag with sulfate-rich bypass dust (1:1) created cementing composites containing a high percentage of ettringite and the strength value reached 18.6 MPa after 90 days, while mixing slag with lime-rich bypass dust (1:1) generated cementitious composites rich in portlandite with 15.1 MPa.

Precast construction materials now frequently use hydrothermal curing to achieve good mechanical characteristics by generating a compact structure^30^. Heat and pressure are applied simultaneously during the process of hydrothermal curing^31^ to accelerate the cementitious materials’ hydration reaction and pozzolanic activity; this kind of curing condition lowers voids and subsequently improves the microstructure of concrete^32^. Hydrothermal curing of geopolymer/AAMs is a crucial step that transforms the hydration gel into zeolite minerals with excellent hydraulic properties^33^. The impact of hydrothermal curing on the slag/fly ash/metakaolin geopolymer was investigated by Wang et al. in 2012. It was noted that depending on the Si: Al: Ca ratio, the aluminosilicate gel (zeolite minerals), analcime, and tobermorite phase were formed. Furthermore, upon hydrothermal curing, most of the manufactured composites’ compressive strength values were above 60 MPa, and the percentage of voids was less than 36%, with a median pore diameter of roughly 12 nm^34^.

Nasr et al.^35^ studied the impact of different curing conditions on the fire resistance of AAS: curing in relative humidity of 33% at 23^o^C, curing in water at 25^o^C, and hydrothermal curing at 2.3 bar/135^o^C for 2 h. The results indicated that the last treatment was the most effective, which catalyzed the production of extra quantities of CSHs, CASHs, and zeolite-like phase in the form of an albite phase (NaAlSi_3_O_8_), which developed the compressive strength. Azarshab et al.^36^ reported that the autoclave curing of AAS at 90^o^C for 24 h induced the transformation of the geopolymer chains into the yagawaralite zeolitic phase (CaAl_2_Si_6_O_160_.4H_2_O). Pre-casting slag/sludge-based alkali-activated pastes were prepared by Ramadan et al.^37^. The outcomes affirmed that autoclaving the pastes at 5 bar for 4 h was enough to achieve high strength (72 MPa). Slag/glass powder waste binders (1:1) were activated by 5wt%NaOH under hydrothermal curing at 4 bar/8hrs. The results confirmed that mesoporous alkali-activated composites’ compressive strength reached 86 MPa due to extra accumulation of CSHs, CAHs, CASHs, and hexagonal zeolites^38^.

One of the most popular dyes in the textile industry, notably for dying denim, is indigo carmine (IC). Because of its remarkable versatility in diagnostic techniques and surgical operations, including microsurgery and gynecological and urological surgeries, it is also utilized in medicine. According to reports, indigo carmine is hazardous to people and can result in several diseases, including gastrointestinal issues, skin irritations, and hypotension^39^. On the other hand, methylene blue (MB) is considered a hazardous cationic dye, which is carcinogenic, mutagenic, and ecologically persistent. It is frequently used as a synthetic dye to color materials in the apparel and textile industries, as well as to color leather and paper. A significant amount of wastewater containing MB and IC dyes is released into the surface and groundwater because of the extent of its industrial use^40^.

Geopolymer has been used to treat wastewater containing industrial colors like cationic dyes because of its unique surface properties (crosslinking, negative charges, and porous structure). Many technologies are being utilized nowadays to remove colors from wastewater; the adsorption method is one of these technologies due to its low energy and cost requirements as well as its potential for large-scale application^41^. Padmapriya et al.^41^ and Alouani et al.^42^ created low-cost adsorbents based on the alkali-activation of slag, fly ash, and bottom ash for effective removing MB. Foamed AAS adsorbents were prepared by Bhuyan et al.^43^. The synthesized pastes were cured at 60^o^C/4hrs. The foaming agent is H_2_O_2_ in the presence of different surfactants as foaming stabilizers. The results indicated that the optimized prepared paste has a compressive strength 2.9 MPa with a specific surface area of 83.3m^2^/g. The optimized adsorbent was designed for MB removal. After 6 h, the MB removal reached 74%, with the initial removal of ~ 100%. The alkali-activation of slag/sludge precursors under hydrothermal curing at 5bars/4hrs created specific cementing-composites with multifunctional applications; the hardened composites possessed high compressive strengths while the powders acted as highly efficient adsorbents for MB removal^44,45^.

Based on the gap in the previous literatures, the novelty behind this work is to manufacture cementing-composites, free from Portland cement, that contribute to carbon sequestration and reduce its environmental problems. Synthesis of these composites was based on the alkali-activation of some industrial solid wastes such as slag, silica fume and cement bypass/dust. The sustainable disposal of these wastes and their recycling for the production of green multifunctional alkali-activated cement pastes are the main targets of our investigation. The functionality of the prepared doughs varied due to the various curing conditions (conventional curing at room temperature and hydrothermal curing at different steam pressures). The compressive strength of the hardened pastes and the dye removal efficiency of some powdered pastes were measured. The phase composition, textural parameters, and morphology of some selected pastes were examined via XRD, TGA/DTG, BET/BJH, and SEM/EDX techniques. The results confirmed that some AAMs can be used for mechanical engineering and environmental purposes to get rid of hazardous MB and IC dyes in wastewater.

## Materials and experimental techniques

### Materials

The starting materials utilized in this investigation are slag, bypass, and silica fume. Ground granulated blast furnace slag (GGBS) was supplied from Lafarge Company, El-Sokhna, Egypt. Bypass (B) was provided from El-Areesh Company, Sinai, Egypt. Silica fume (S) was delivered from Sika Company, El-Qalyubia, Egypt. The activator used is sodium hydroxide (NaOH of purity ≥ 98%)) that was supplied from Alfa Aromatic Company, Cairo, Egypt. The chemical oxide compositions of GGBS, B, and S were analyzed via the X-ray fluorescence technique (XRF, model PW-1400, Xios) and given in Table 1. Methylene Blue (MB) and Indigo Carmine (IC) dyes were supplied from Alfa Aromatic Company. Table 2 displays general characteristics of these dyes.   

**Fig. 1:**
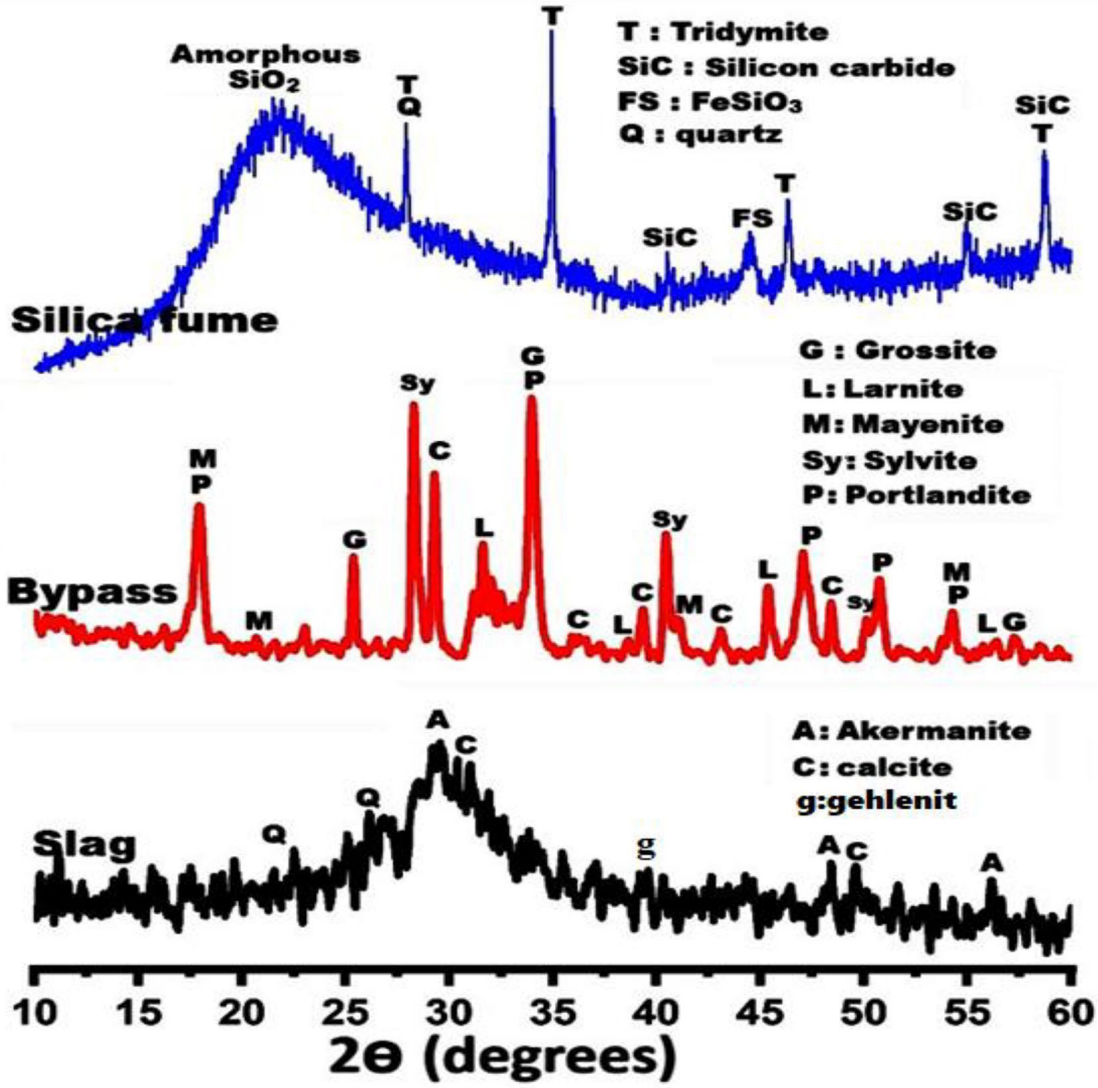
XRD-patterns of raw materials.

**Fig. 2:**
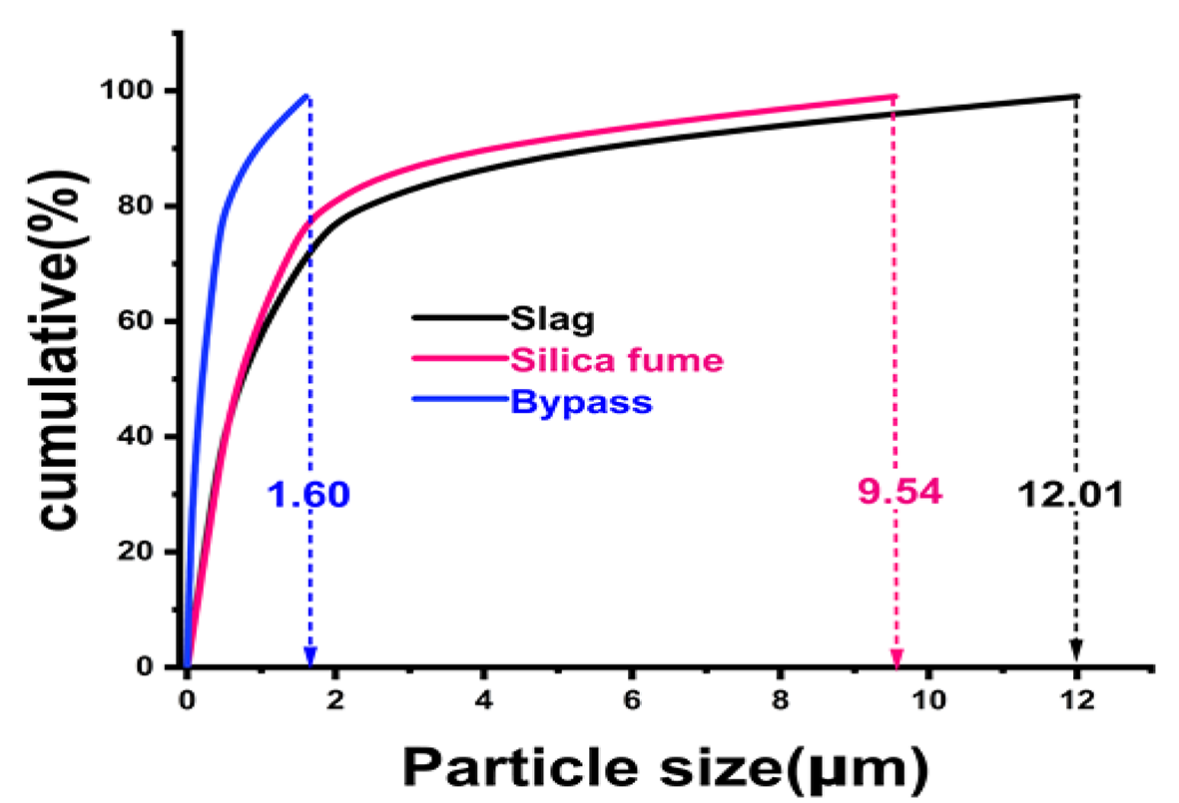
Particle size distribution curves of raw materials.

The X-ray diffraction (XRD, model Xpert-2000, Philips) in Fig. 1 demonstrates the phase composition of GGBS, B, and S. The XRD pattern of GGBS displayed a wide hump (2θ = 25^◦^ − 37^◦^) comprising phases of akermanite (Ca_2_Mg[Si_2_O_7_], PDF# 01-079-2424), gehlenite (Ca_2_Al[AlSiO_2_], PDF# 01-077-1113) and calcite (CaCO_3_, PDF# 00-005-0586). This wide hump reveals the high amorphousity of slag. While the distinct phases detected for bypass specimen are grossite (CaAl_4_O_7_, 2θ = 25.41, 34.07, 57.27◦), larnite (Ca_2_SiO_4_, 2θ = 31.59 ,38.67 ,45.35 ,56.41◦), mayenite (12 CaO·7Al_2_O_3_, 2θ = 17.93 ,20.73 ,41.07 ,54.29◦), sylvite (KCl, 2θ = 28.27, 40.43, 50.15◦), portlandite (Ca (OH)_2,_ 2θ = 17.93, 34.07, 47.03, 50.81, 54.29◦), calcite (CaCO₃, 2θ = 29.31, 36.01, 39.27, 43.07, 48.51◦). XRD of silica fume indicates a high broadness peak located at 2$\:\theta\:$ = 15-35^o^ with a maximum at 2$\:{\uptheta\:}$ = 22.5^o^ that is assigned to the high content of glassy amorphous silica, in addition to some crystalline peaks that are attributed to the presence of quartz, silicon carbide, and tridymite phases, (Fig. 1). As clarified in Fig. 2, the particle size distribution of raw materials using PSD: PSS-NICOMP, N3000 analyzer indicates that the D_50_ of GGBS, B, and S particle sizes is less than 12.01, 1.60, and 9.54 μm, respectively. At the same line, the Blaine surface area (BSA: Humboldt, H-3810) analyzer referred that the fineness of GGBS, B, and S powdered samples is 3500, 5500, and 5750 cm^2^/g, respectively.

**Table 1:** Chemical oxide compositions of various raw materials (mass, %).

Materials	SiO_2_	Al_2_O_3_	Fe_2_O_3_	CaO	MgO	SO_3_	Na_2_O	K_2_O	Cl	LOl
GGBS	50.01	14.11	0.78	24.50	6.32	1.78	0.52	0.86	0.61	0.51
B	12.33	3.54	1.98	54.62	0.52	4.24	0.54	6.12	4.38	
S	92.00	1.39	1.41	0.80	0.89		1.00	1.00		1.51

**Table 2:** General characteristics of the IC and MB dyes.

Characterization	Type of used dye	
	MB	IC
Charge	Cationic dye	Anionic dye
Molecular structure		
Chemical formula	C_16_H_18_ClN_3_S	C_16_H_8_N_2_Na_2_O_8_S_2_
Molecular weight (g/mol)	319.9	466.36
ℷ_max_(nm)	668	609

### Experimental techniques

#### Preparation of alkali-activated cement pastes

In this study, five alkali-activated cement pastes were prepared. The composition of each paste is displayed in Table 3. Before the paste preparation, the dry mixture of each composition was prepared by mixing the required weight of raw materials using a ball mill for 3 h to attain complete homogeneity. The 1st dry mixture contained 800 g slag (Control, C). The 2nd dry mixture (C-20B) contained 640 g slag + 160 g bypass. The 3rd dry mixture (C-10B10S) contained 640 g slag + 80 g bypass + 80 g silica fume. The 4th dry mixture (C-30B) contained 560 g slag + 240 g bypass. The 5th dry mixture (C-20B10S) contained 560 g slag + 160 g bypass + 80 g silica fume. Each dry mixture was activated by 5 M NaOH solution (48 g NaOH dissolved in 240 ml water). The ratio of mixing water to the cementitious solids (W/S) is 0.3. The alkaline solution was gradually cooled to room temperature (25^o^C). The use of this concentration was based on the consistency of the prepared doughs, good activation of the granules, and the avoidance of economic costs.

**Fig. 3:**
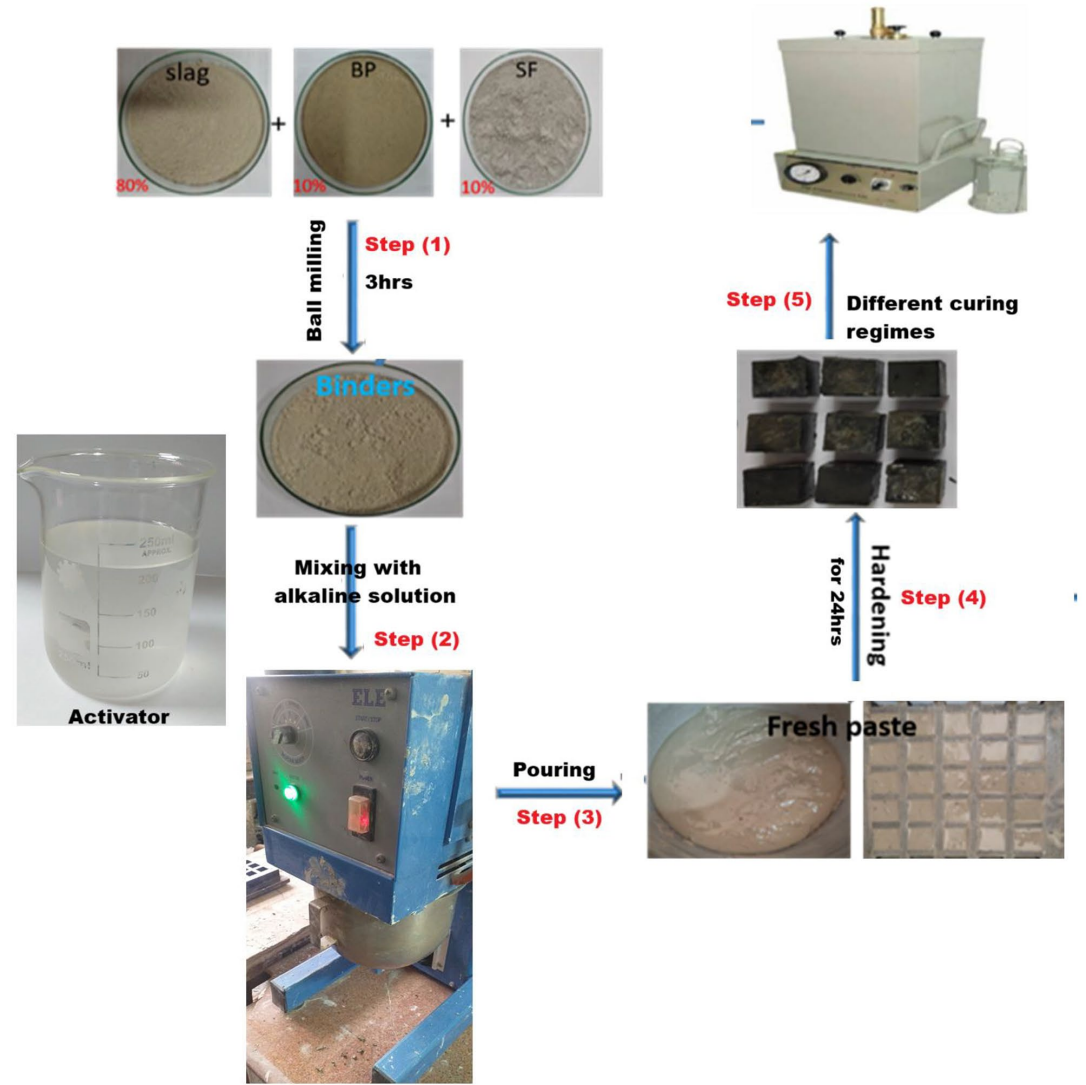
Schematic diagram displays the preparation steps of alkali-activated-cement pastes.

After that, each dry mixture was mixed with the prepared alkaline solution using a Hobart automatic mixer for 3 min to get a homogeneous and good workable paste^35,36^. The prepared paste was poured into a cubic mold (25*25*25 mm) and then left at high humidity (98 ± 2%) for 24 h in the humidity chamber; this time is enough for the paste to harden. The hardening process for the cubic pastes depends on the type of treatment/curing. Hence, two curing regimes were applied: (i) conventional curing in high humidity (≈ 98 ± 2%) at 25^o^C for 1, 3, 7, 28 and 90 days. (ii) curing under hydrothermal conditions in a high-pressure autoclave (model ELE-32, Controls) at 7 bar/178◦C for 1, 4, 8, and 12 h. On the other hand, different pressures (4 bar/153◦C, 7 bar/178◦C, and 10 bar/198◦C) were applied for 4 h. Our choice of these conditions is based on previous literature and simulation of industrial reality, which emphasizes obtaining good mechanical results. Figure 3 displays the preparation steps of alkali-activated cement pastes.

**Table 3:** Mix design alkali-activated cement pastes (mass, %).

AAMs	GGBS %	B %	S %	W/S ratio	NaOH solution (M)
C	100			0.3	5
C-20B	80	20		0.3	5
C- 10B10S	80	10	10	0.3	5
C-30B	70	30		0.3	5
C-20B10S	70	20	10	0.3	5

#### Mechanical testing and characterizations

The compressive strength of the hardened pastes was measured according to ASTMC109M-20b^37^. A compression testing machine was used (60 tons maximum load, Controls). Four cubic pastes were tested to evaluate the average compressive strength at each curing time (1, 3, 7, 18, and 90 days under conventional curing). On the other hand, the average compressive strength of autoclaved cubes was also tested at each steam pressure or autoclaving time. The results indicated that the control pastes and C-10B10S pastes possessed good mechanical properties after 28-days of normal curing and under autoclaving conditions at 7bars/4hrs, so these alkali-activated cement pastes (C and C-10B10S) were selected to examine their formed phases, morphology and textural characteristics. X-ray diffraction (XRD) and thermogravimetric analysis (TGA/DTG, NETZSCH, STA449-F5) techniques were conducted to examine the formed phases inside the structures of AAMs. A thermo-Scientific scanning electron microscope (SEM/EDX) has been used to study the morphological features of the hydration products formed. N_2_-adsorption/desorption technique (BELSORP-miniX (S/N: 151, Version 1.0.9.0) was applied for some selected pastes to identify the BET surface area, monolayer capacity, total pore volume, and BJH-average pore diameter.

#### Dyes removal

For evaluation of the efficiency of some selected hydrothermally treated pastes (C and C-10B10S) to be used in the treatment of wastewater, the following test was performed:1000 ppm solution of each selected dye (MB or IC) was prepared as a stock solution, and this solution was progressively diluted as required for various tests. Adsorption tests in batch mode were performed by adding 0.5 g of the sample to a 100-ml stoppered glass holding 50 ml of a 20-ppm of IC or MB dye solution and stirring it on a thermostatic orbital shaker. The effect of pH (2–12) in a series of batch adsorption studies. Samples were stirred at 250 rpm for 2 h to ensure that adsorption equilibrium was attained in each batch adsorption test. Calibration curves were constructed prior to the measurement using the standard MB or IC solution with established concentrations. The following formula is used to calculate the dye clearance percentage:

1 $$\:\text{R}\text{e}\text{m}\text{o}\text{v}\text{a}\text{l}\:\text{p}\text{e}\text{r}\text{c}\text{e}\text{n}\text{t}\text{a}\text{g}\text{e}\left(\text{\%}\right)=\frac{\text{C}^\circ\:-\text{C}\text{t}}{\text{C}^\circ\:}\text{*}100$$

where C° and Ct (mg/l) are the liquid-phase concentrations of dye at initial and any time (t), respectively^45^.

The point of zero charge (pH_PZC_) of C and C-10B10S powders was determined to understand the effect of pH values on the removal efficiency of the prepared specimens. For this test, twelve glass bottles of 50 ml were prepared containing 0.5 g of alkali-activated cement powder. This powder was suspended in a 50 ml solution of KCl (0.1 M). Six bottles in the presence of MB and other six bottles in the presence of IC. The initial pH values applied are 2,4,6,8,10,12 (adjusted using 0.1 M NaOH or/and HCl). After that, the suspensions were automatically shaken overnight, and the pH was determined as final. The $\:\varDelta\:$pH was computed as follows:

2 $$\:\text{p}\text{H}\:\text{f}\text{i}\text{n}\text{a}\text{l}-\text{p}\text{H}\:\text{i}\text{n}\text{i}\text{t}\text{i}\text{a}\text{l}$$

∆pH versus $\:\text{p}\text{H}\:\text{i}\text{n}\text{i}\text{t}\text{i}\text{a}\text{l}$ has been plotted. The point zero charge (PZC) is recorded from the vertical projection of the curve^44^. The obtained results were further supported by measuring the zeta potential, which was determined by measuring the electrophoretic light scattering with Litesizer 500 (Anton Paar GmbH, Austria). The voltage and processed runs were adjusted automatically, and the measurements were done using the Omega cuvette.

## Results and discussion

### Compressive strength

**Fig. 4:**
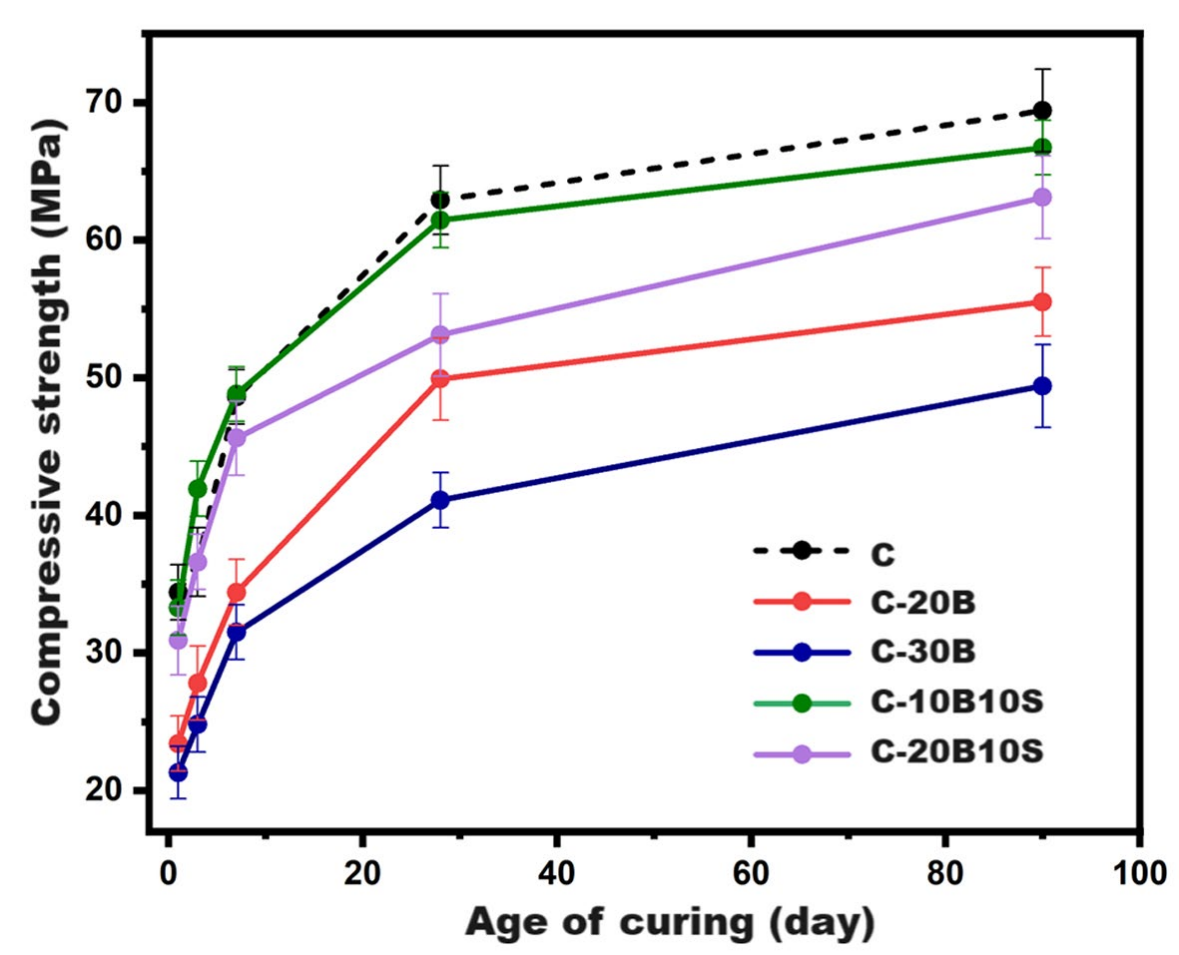
Compressive strength values for different alkali-activated composite pastes under normal curing condition up to 90-days.

Figure 4 displays the compressive strength values of the prepared NaOH-activated pastes under high humidity curing ($\:\sim$100%) up to 90 days: control (C, 100% GGBS), C-20B, C-30B, C-20B10S, and C-10B10S. The general trend for all pastes is a significant increase in the strength value up to 28 days, followed by a gradual increase up to 90 days of curing, which indicates progressing the hydration/alkali-activation reaction of most unreacted grains (slag, bypass, or silica fume) during the first month to generate strength-giving phases such as calcium silicate hydrates (CSHs), calcium-alumino-silicate hydrates (CASHs), calcium aluminate-hydrates (CAHs), sodium-alumino-silicate-hydrates (NASHs), in addition to hydrotalcite (Ht)^6,7,17,38,46^, and the formation of these phases was confirmed using XRD and TGA/DTG techniques. As clarified in Fig. 4, replacing slag with bypass up to 30% induces a strong deterioration in the strength values of C-20B and C-30B pastes at all curing ages. Compared with the control pastes, the compressive strength of C-30B pastes decayed by 38, 34.7, and 28.8% at 1, 28, and 90 days, respectively. The decline in the strength values is attributed to the strong dilution/reduction of akermanite (2CaO.MgO.2SiO_2_) and gehlenite (2CaO.Al_2_O_3_.SiO_2_) phases as the main active precursors, which are responsible for the formation of many different strength-giving hydrolytic products. On the other hand, the high content of bypass led to the existence of large quantities of inactive/highly crystalline phases such as larnite (β-2CaO.SiO_2_), grossite (CaO.2Al_2_O_3_)^47^, and mayenite (12CaO·7Al_2_O_3_) which partially disintegrated very slowly by alkali. These outcomes are agreed with the results of Hashem et al. (2024), who confirmed that increasing the content of bypass caused a large reduction in the content of reactive silica, which considered the main factor behind the formation of Si-O-Si and Si-O-Al bonds inside the structure of AAMs^48^. Also, Hassani and Zhang (2022) pointed out that the high replacement of bypass led to the availability of large quantities of Ca^2+^ and Na^+^ concentrations, which induce the formation of N–(C)–A–S–H gel of low hydrolytic nature^49^. Significant developments were observed in the compression resistance values of C-20B and C-30B when replacing 10% of the bypass with 10% silica fumes (S) and it was found that the pastes containing 80%GGBS+10%B + 10%S (C-10B10S) were the closest mix to the control sample (C). As mentioned in the previous literature, highly amorphous silica fume played an important role in boosting the strength values for the following reasons: (I) it acted as an effective micro-filler that mechanically reinforced the microstructure of cementitious composite, (II) it worked as nucleation sites which induce alkali-activation reaction and catalyze dissolution of unreacted akermanite and gehlenite phases, and (III) pozzolanic material which chemically reacted with different unreacted phases of bypass such as portlandite, grossite and mayenite^50,51^ to generate extra amounts of CSHs, CASHs, and CAHs.

Table 4 Demonstrates the obtained compressive strength values for various specimens subjected to hydrothermal curing at 7 bar/178^o^C. The strength values displayed a continuous fast development with time of steam pressure curing (from 1 to 12 h) for all pastes (C, C-20B, C-30B, C-10B10S, C-20B10S). These results are assigned to the acceleration of the alkali-activation reaction of the active phases (containing amorphous silica and/or alumina) present in the used precursors (GGBS, B, S), which promoted the formation of huge quantities of various products, namely as CSHs, CASHs, CAHs, and NASHs. Evidently, regarding the economic, environmental and performance points of view, 4 h can be considered the optimum curing time for nearly all prepared pastes as about 80–90% of strength was achieved during this period. Moreover, many previous literatures considered this time sufficient for forming zeolite-like phases that contribute to the development of mechanical performance^37,38,52–54^.

**Table 4:** The compressive strength values for various specimens cured at 7 bar/178^o^C.

Time of autoclaving (hr)	Compressive strength (MPa)				
	C	C-20B	C-30B	C-10B10S	C-20B10S
1	56.7	36.3	31.4	60.3	52.2
4	80.0	54.2	45.4	72.6	63.7
8	83.3	60.6	47.1	75.0	69.6
12	92.8	71.2	51.0	78.7	72.4

**Fig. 5:**
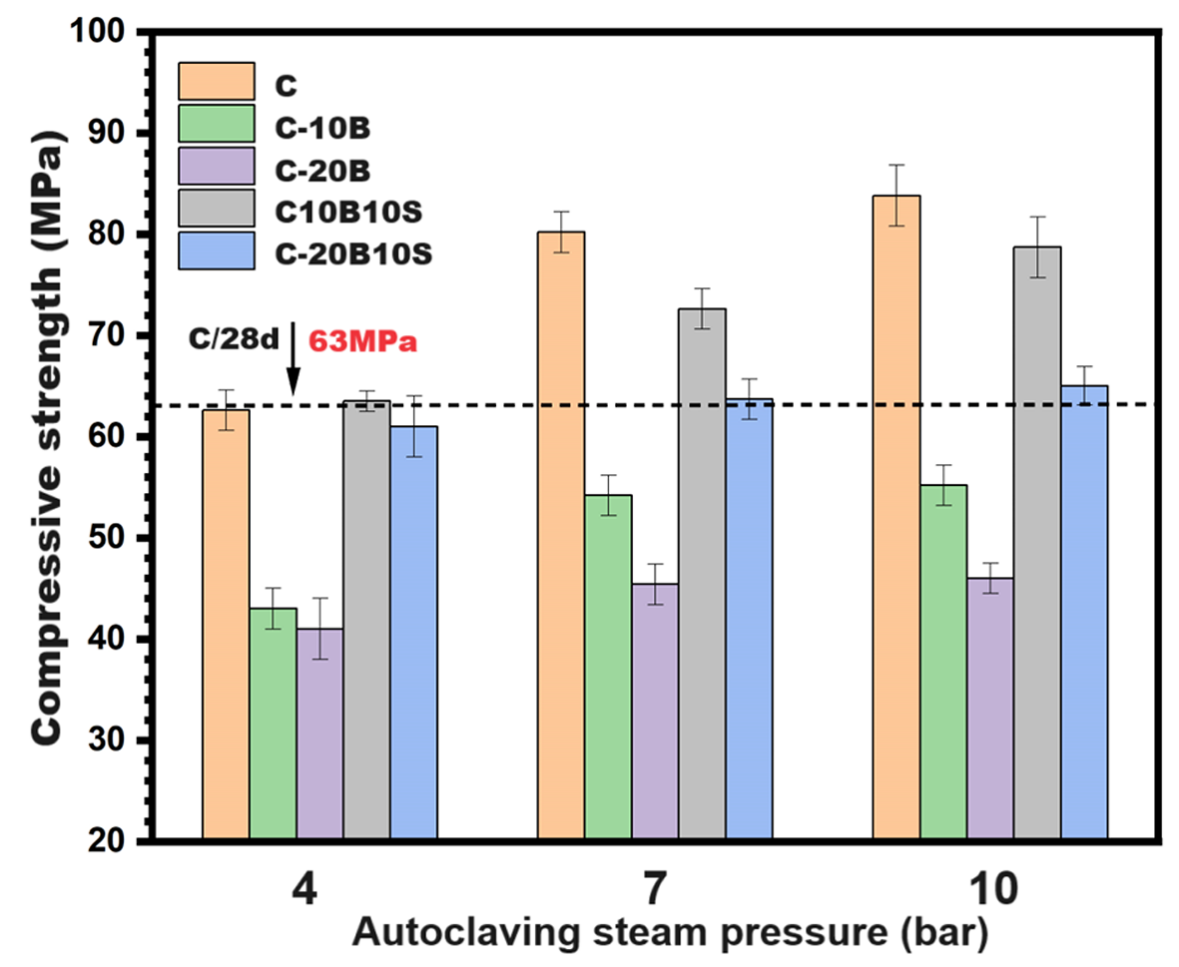
Compressive strength values for different alkali-activated composites subjected to hydrothermal curing at different steam pressures for 4 h.

Figure 5 illustrates the compressive strength values of all mentioned pastes treated at different steam pressures (4, 7, and 10 bar) for 4 h. The figure demonstrates a progressive development in the compression resistance of the formed geopolymers (compressive strength) with elevating the steam pressure from 4 to 10 bars for all autoclaved pastes (C, C-20B, C-30B, C-10B10S, C-20B10S). Evidently, elevating pressure led to the acceleration of the hydration reaction or the condensation rate to generate large quantities of hydration products (CSHs, CASHs, CAHs, NASHs) within four hours^37,52^. As shown in the figure, the autoclave curing of C-10B10S pastes at 4 bar for 4 h was sufficient to reach the compressive strength reading (63 MPa) of the control sample (C) at 28 days of normal treatment and this value was enhanced to 72 and 79 MPa at 7 and 10bars, respectively. The great development in the mechanical performance of all prepared pastes (specially C-10B10S composite) under hydrothermal curing is attributed to the formation of highly compacted structures based on: (i) transformation of amorphous NASHs into zeolitic structures^55^ as detected via XRD and SEM/EDX, (ii) pore structure rearrangement^56^ from macro to meso-pores as affirmed by BET/BJH-model, (iii) generation of extra amounts of strength-giving phases especially the hydrogarnet phase (C_3_AH_6_) which detected by XRD and SEM/EDX techniques.

### Statistical analysis

ANOVA analysis was conducted to define the key factors in the prepared mixes-design that affect the compression strength of alkali-activated cement pastes. As displayed In Table 5, there are important outputs that evaluate the statistical significance, such as F statistics (F), F critical (Fcrit), and the null hypothesis (p-value). If Fcrit < F and p-value < 0.05; this indicates the significant importance of mix-design’ variations^57–59^. Table 5 demonstrates that the effect of different mix-design constituents at 1 and 28 days of normal curing significantly impacted the compressive strength values. Change from normal to the autoclave treatment at 7 bar/4hrs had the greatest significance (p-value = 3.79*10^− 8^). In the same line, the effect of autoclave curing time (2, 4, 8, 12 h) at 7 bar for C-10B10S and the effect of autoclave curing pressure (4, 7, 10bars) for 4 h for C-10B10S have significant impacts on the compressive strength.

**Table 5:** ANOVA results for compressive-strength value.

Factor	Specimens Or Curing condition	*SS*		*df*		*MS*		*F*	*P-value*	*F crit*	Significance criteria (*P* < 0.05)
		Between Groups	Within Groups	Between Groups	Within Groups	Between Groups	Within Groups				
Effect of different mix-design at normal curing (1-day)	C	423.996	43.72	4	10	105.999	4.372	24.244	3.95E-05	3.478	Significant
	C-20B										
	C-30B										
	C-10B10S										
	C-20B10S										
Effect of different mix-design at normal curing (28-days)	C	952.464	64.5	4	10	238.116	6.45	36.917	5.83E-06	3.478	Significant
	C-20B										
	C-30B										
	C-10B10S										
	C-20B10S										
Effect of different mix-design at autoclave curing (7 bar/4hrs)	C	1717.224	40.44	4	10	429.306	4.044	106.158	3.79E-08	3.478	Significant
	C-20B										
	C-30B										
	C-10B10S										
	C-20B10S										
Effect of autoclave curing time at 7 bar for C-10B10S	1 h	571.95	25.5	3	8	190.65	3.187	59.811	8.03E-06	4.066	Significant
	4 h										
	8 h										
	12 h										
Effect of autoclave curing pressure for 4 h for C-10B10S	4 bar	372.86	28.1	2	6	186.43	4.683	39.807	0.00034	5.143	Significant
	7 bar										
	10 bar										

### Phases identification

#### X-ray diffraction

**Fig. 6:**
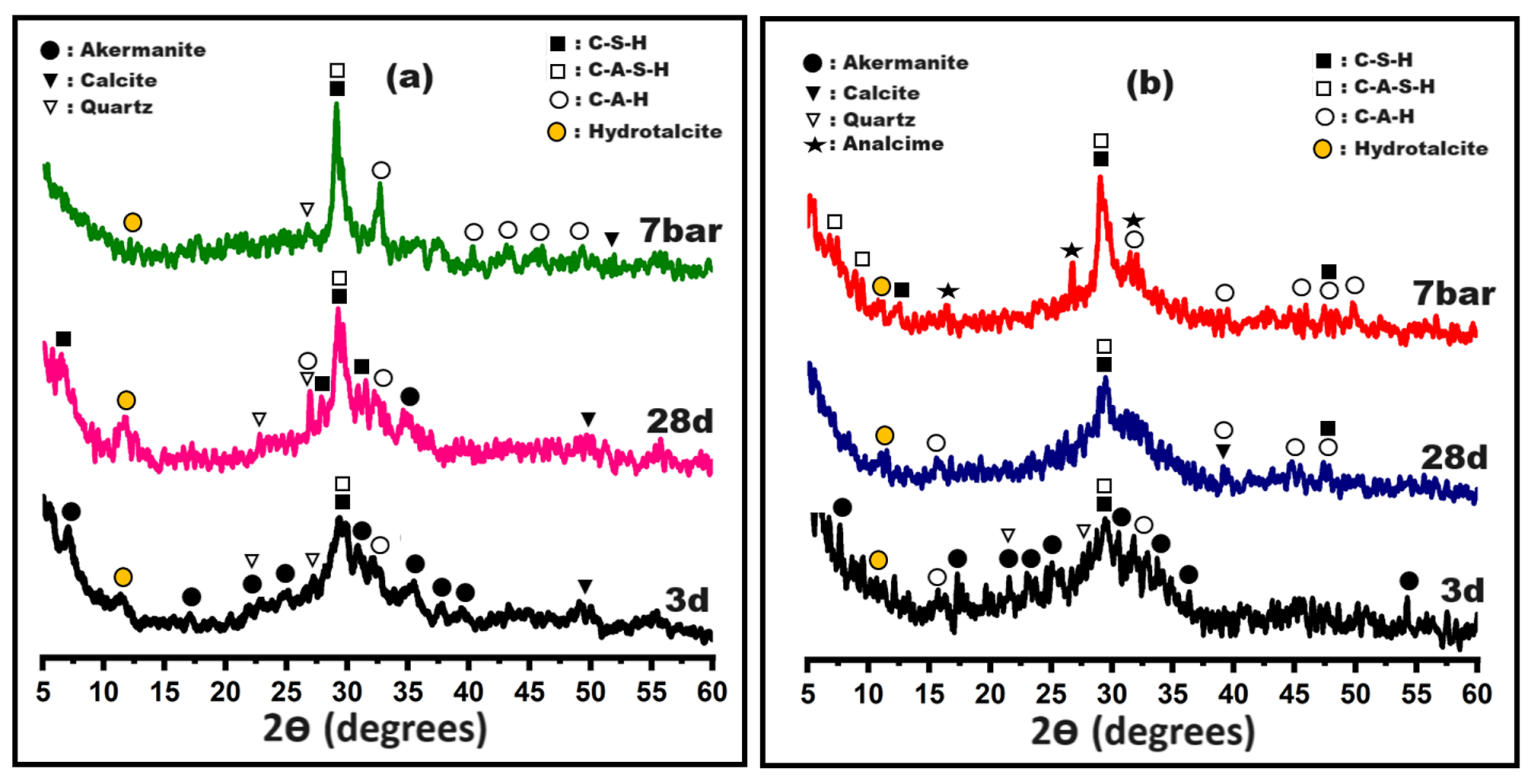
XRD-patterns for **(a)** Control and **(b)** C-10B10S composite pastes at different curing conditions.

From sustainability, green environment, and performance points of view, C and C-10B10S pastes were selected to examine their formed phases under different curing conditions: high humidity up to 28 days at room temperature and hydrothermal curing at 7 bar for 4 h. According to what was reported in previous literature, the type of created phases and their crystallization strongly affect the mechanical performance/microstructure of the prepared alkali-activated cement pastes^52^. Therefore, the phases formed can be tracked through the XRD technique. Figure 6-a displays XRD-patterns of the control specimens (C) at 3 and 28 days of normal curing in addition to hydrothermal curing at 7 bars. The figure confirms that the three-day alkaline activation was insufficient for the dissolution of most akermanite (PDF# 96–901–3639). However, hydrotalcite (PDF#96-210-2793), ill-crystalline calcium silicate hydrates/tobermorite gel (CSH, PDF#00-002-0068), calcium alumino-silicate-hydrates (CASH, PDF# 00–002–0047), and amorphous calcium-aluminate-hydrate (CAH, PDF# 96-210-3046) are detected at 2$\:\theta\:$= 11.37, 29.29, 29.39, and 32.01^o^, respectively. Furthermore, the broad band at 2$\:\theta\:$=20-37^o^ is a good indication for forming sodium alumino silicate hydrate gel (NASH)^60,61^. By continuing the normal treatment for about a month, we noticed that most of the akermanite disappeared due to its being dissolved and transformed into crystalline CSH and CASH, in addition to hydrotalcite and CAH. Changing the nature of the treatment to hydrothermal curing led to the disappearance of most quartz and the formation of highly crystalline hydrogarnet (CAH, C_3_AH_6_, PDF#96-900-1086), highly crystalline CSHs (PDF#00-034-0002) and CASHs (PDF#96-900-5060). It is worth noting that the appearance of hydrogarnet with an increase in the degree of crystallinity of the formed phases help in the formation of strong networks that support the porous structure and fill the large interstitial spaces, which contribute to the development of compressive strength^37,55,62^. Figure 6-b displays XRD-patterns of C-10B10S pastes under normal and hydrothermal curing. The same mentioned phases were detected under normal curing, but a new zeolite phase (analcime, PDF#96–900–4013) was observed at 7 bar with disappearance of the distinct broadness. Therefore, the phase transformation of amorphous NASH gel into highly crystalline analcime may be one of the important reasons for improving the mechanical efficacy of this composite.

#### Thermal analysis (TGA/DTG)

**Fig. 7:**
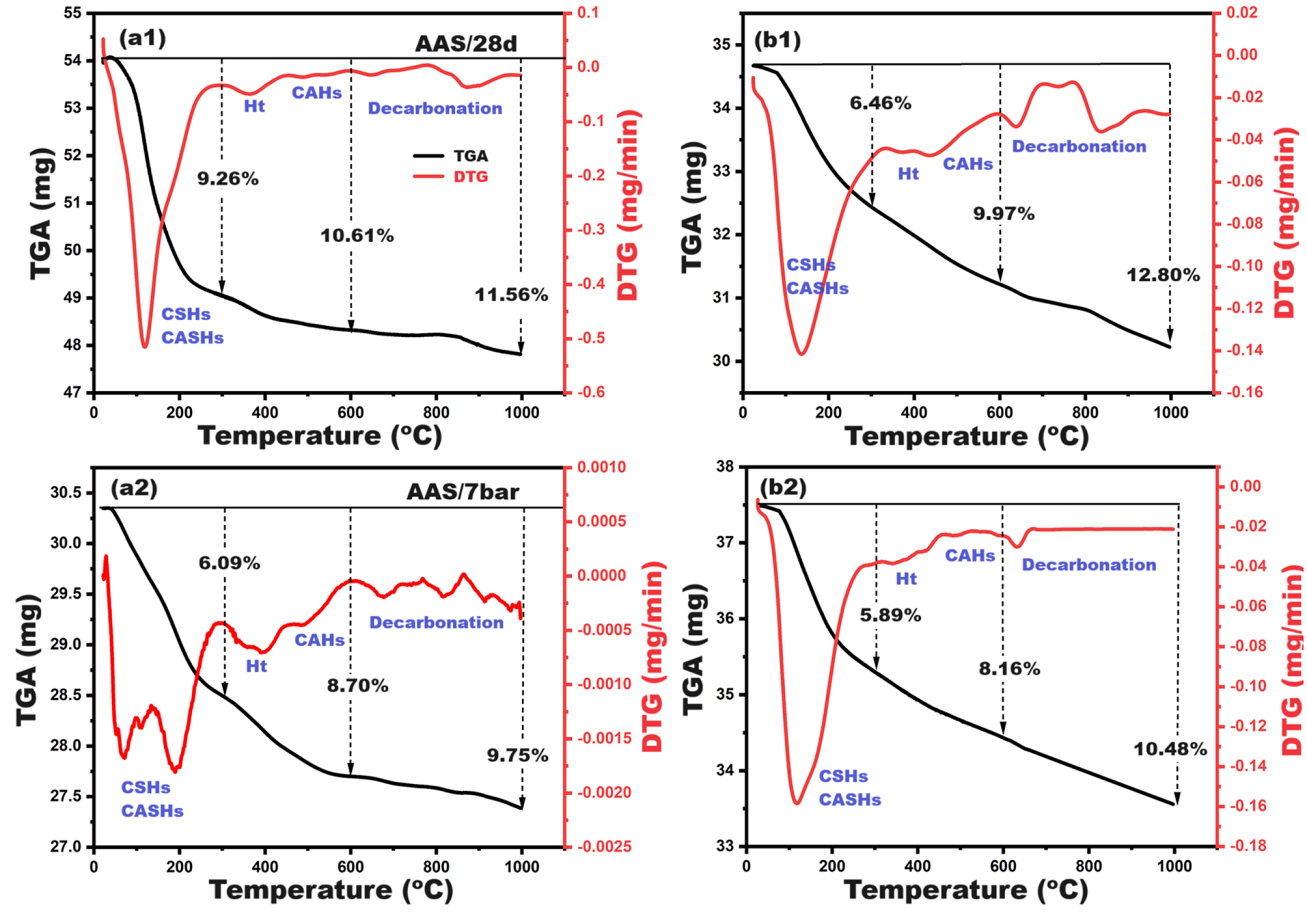
TGA/DTG thermogram for control (**a1**, **a2**) and C-10B10S (**b1**, **b2**) pastes at different curing conditions.

Thermogravimetric analysis is the most effective technique for determining the concentration of phases formed resulting from the alkali activation process, by tracking the loss in weight with heating up to 1000 degrees. Figure 7 illustrates TGA/DTG thermograms for control (C) and C-10B10S composite pastes under normal and autoclave curing. Figure 7-a1 demonstrates TGA/DTG curves at 28-days of normal curing (high humidity at room temperature). Three successive mass losses (TGA%, black curve) were detected at different temperature zones, which were accompanied by five endothermic peaks (DTG, red curves). The first loss (9.26%) was located at 50-300^o^C and harmonized with a strong endothermic peak. This behavior is attributed to dehydration/deterioration of the formed calcium-silicate-hydrates (CSHs of high-water content) and calcium-alumino-silicate-hydrates (CASHs)^63^. The second loss (10.61–9.26 = 1.35%) is located at 300-600^o^C and paralleled by two weak endothermic peaks, which confirm the creation of hydrotalcite and calcium aluminate hydrates, especially hydrogarnet (C_3_AH_6_)^7^. The last loss (11.56–10.61 = 0.95%) was observed at 600-1000^o^C due to the thermal decomposition of different carbonated phases^16,64^. As reported in previous literature^52^, the strength-giving phases, such as CSHs, CASHs, C3AH6, and hydrotalcite, were evaluated by 10.61% inside the control’s structure under normal curing. Replacing slag with 20% solid wastes (C-10B10S) did not cause a significant change in this percentage, but a slight decrease was assigned from 10.61 to 9.97% (Fig. 7-b1). Therefore, the mechanical properties were comparable for the two pastes: 63 and 61.5 MPa for C and C-10B10S, respectively, after 28-days. On the other hand, a significant decrease was recorded in the TGA% under hydrothermal curing for the two mentioned composites, however the corresponding strengths were high. It was found that TGA %, up to 600^o^C, for C and C-10B10S autoclaved pastes were 8.7 and 8.16% [Fig. 7 (a2, b2)] and the measured compressive strength at 7 bar was 80 and 72 MPa, respectively. This trend affirms the creation of highly crystalline hydration products of low-water content^65–67^ and/or the transformation of some alumino-silicates into thermal stable zeolitic networks^37,68^. These outputs can be benefitted by interpreting the mechanical performance of alkali-activated cement as well as the removal efficiency of dyes. The increase in the amounts of crystalline hydrates, in addition to the formation of zeolites, contributes significantly to the formation of mesoporous densified structures with high compressive strength and high adsorption capacity, so these types of materials can be used as filters for wastewater treatment as discussed in detail in Sect. 3.6.

### Surface characterization

**Fig. 8:**
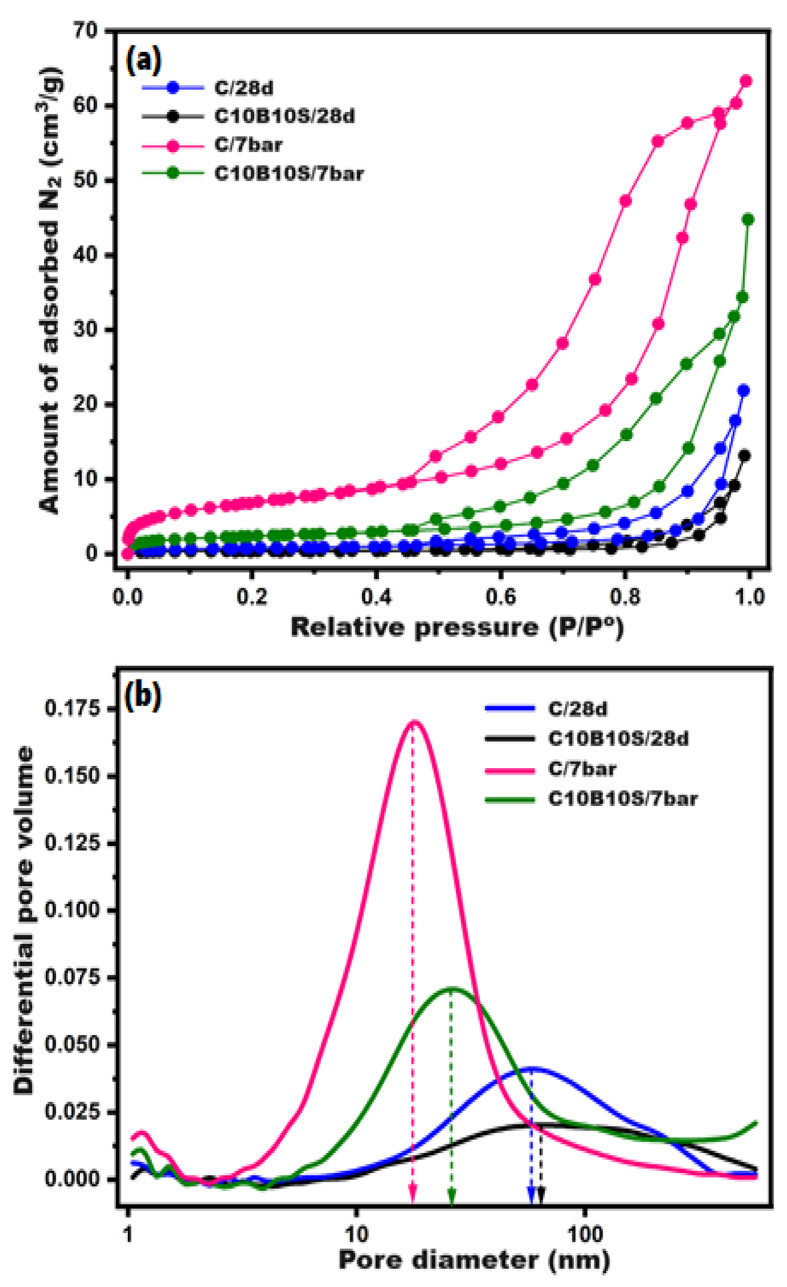
**(a)** N_2_-adsorption/desorption isotherms and **(b)** BJH-pore size distribution curves for the selected composites at different curing conditions.

Discovering the fine porous structure of the prepared composites contributes strongly to elucidating the mechanical performance. C and C-10B10S were selected to examine their textural parameters (specific surface area, monolayer capacity, total pore volume, and maximum pore diameter) by means of BET/BJH models. These models reported that materials are classified into three categories: micro-porous (pore size < 2 nm), meso-porous (pore size = 2–50 nm), and macro-porous (pore size > 50 nm)^56,69^. It was found that shifting to microporosity encouraged the creation of high-densified/or reinforced geometries^38^. Figure 8 (a, b) illustrates N_2_-adsorption/desorption isotherms and pore size distributions of C and C-10B10S pastes under normal (28-days) and hydrothermal (7bars/4hrs) treatments. As indicated in Fig. 8-a, the obtained results evidently revealed that autoclave curing significantly impacts the isotherm type. According to the IUPAC classification, the two selected pastes under normal curing indicated Type III isotherms, while hydrothermal curing generated Type IV/H3 isotherms. This turning point affirms the structure shifting from macro to meso-porous^70^. Moreover, the control sample (C) at 7 bar exhibited the highest monolayer adsorption capacity, whatever the relative pressure; this behavior refers to the highest meso-porosity and highest surface area^71^ as compared with other pastes (C/28d, C-10B10S/28d, and C-10B10S/7 bar).

Figure 8-b displays the pore size distributions of the selected pastes. It was detected that the BJH-maximum pore diameter (dpmax) for C/28d, C-10B10S/28d, C/7 bar, and C-10B10S/7 bar were 59.47, 61.42, 18.38, and 35.89, respectively. The results showed that the hydrothermal curing at 7 bars contributed to the rearrangement of the porous structure from wide to medium/mesopores, and this change is attributed to two main reasons. The first one is the production of highly crystalline hydration products (CSHs, CASHs, and C_3_AH_6_) deposited within the wide pores to form tight geometric networks^38,58^. The second reason is converting a certain portion of aluminosilicates and quartz into zeolite networks (analcime) of high adsorption capacities^37,45^. Table 6 shows surface analysis using BET and BJH models for the selected pastes. The obtained data confirmed that hydrothermal curing contributes to developing all histological parameters, and the control paste at 7 bars possessed the highest specific surface area (S.S.A. = 24780 cm^2^/g), highest pore volume (Vt = 0.097 cm^3^/g), highest monolayer capacity (Vm = 5.693 cm^3^/g), and lowest pore diameter (18.38 nm). This data matches the compressive strength values as the strength order was C/7 bar > C-10B10S/7 bar > C/28d > C-10B10S/28d.

**Table 6:** Surface analysis using BET and BJH models for selected pastes at different curing conditions.

Selected paste	Curing condition	S.S.A (cm^2^/g)	Vm (cm^3^/g)	Vt (cm^3^/g)	dpmax (nm)	Isotherm type
C	28-days	2810	0.646	0.033	59.47	III
	7 bars	24,780	5.693	0.097	18.38	IV/H3
C-10B10S	28-days	1370	0.315	0.019	61.42	III
	7 bars	8300	1.907	0.056	35.89	IV/H3

### Morphology

**Fig. 9:**
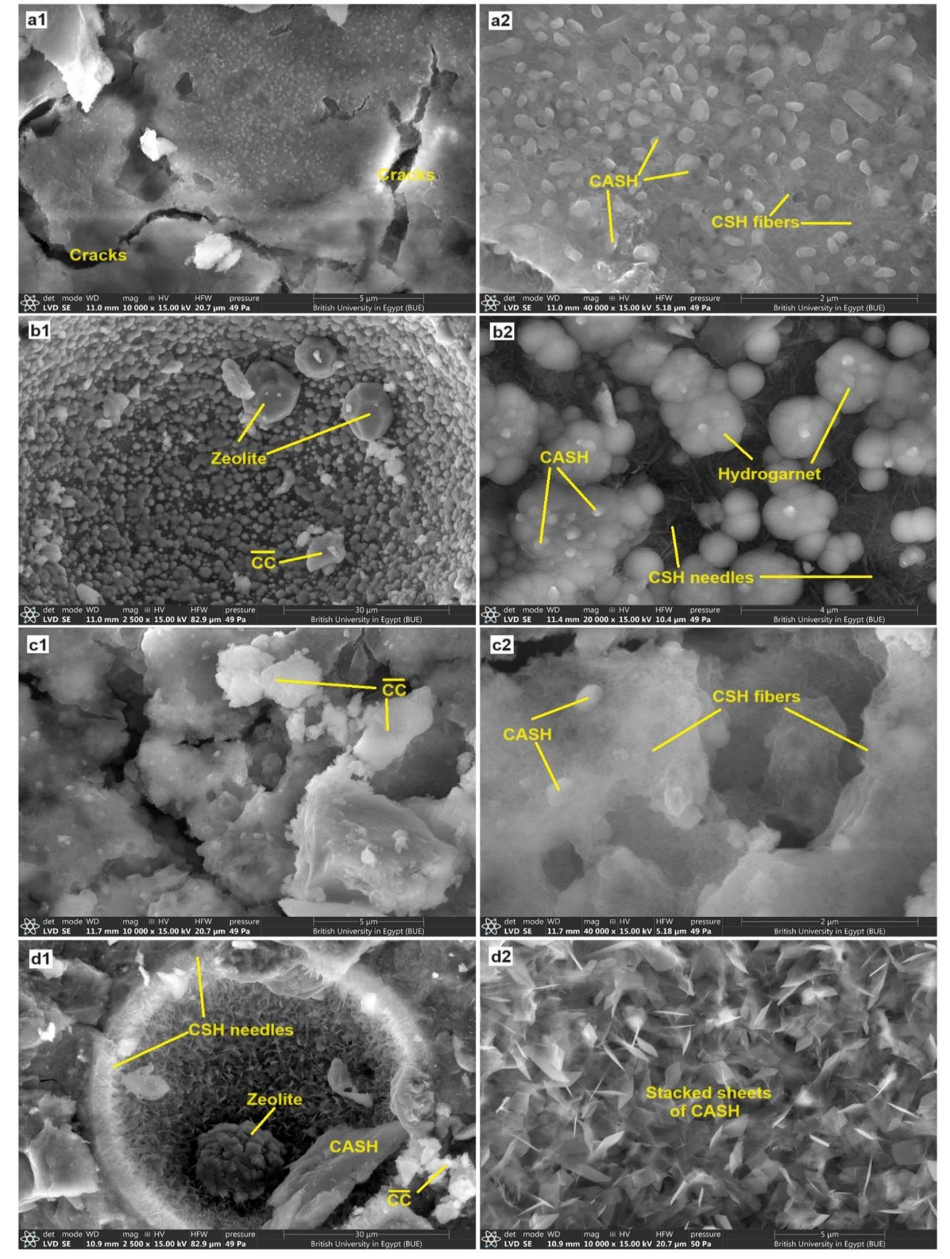
SEM images for selected pastes: (**a1**, **a2**) C/28days, (**b1**, **b2**) C/7 bar, (**c1**, **c2**) C 10B10S/28days.

The mechanical performance of the formed alkali-activated pastes is mainly correlated to the types and morphological nature of the formed phases. So, the SEM/EDX technique was utilized to identify the morphology of the formed phases in the selected specimens (C and C-10B10S). Previous literature has proven that the nature of the microstructure and the shape of the hydration products (CSHs, CASHs, and CAH) vary depending on the curing conditions, which is reflected in the mechanical efficiency of the hardened composites^16,17,44,64,72^. Figure 9 displays SEM images of C and C-10B10S at diverse curing regimes. Figure 9 (a1, a2) shows the microstructure of C paste after 28-days of normal curing. CSH fibers, tobermorite gels, and nano/micro flakes of CASHs were generated after the alkali-activation of slag under normal curing (humidity $\:\approx\:$98% at ~ 25^o^C). Despite the appearance of significant cracks, the creation of these phases strongly supported the microstructure, so the compressive strength of the control pastes achieved 63 MPa. Curing the control pastes at 7 bars/4hours caused radical changes in the resulting phases and pore structure as the micro-cracks disappeared or healed in addition to phase transformations into octahedral zeolite (ANA-analcime)^73,74^, hexagonal plates of CASHs^69^, needles of CSHs, and huge quantities of fused tetrahedral shapes of hydrogarnets^15,75^, Fig. 9 (b1, b2). Increasing the degree of crystallinity of the formed phases contributed strongly to the tightness of the porous structure^76^, and thus the mechanical resistance reached 80 MPa.

Figure 9 (c1, c2) demonstrates the microstructure of C-10B10S paste after 28-days of normal curing. Cracks, calcite, CSH gel, and chips of CASH have been generated. The autoclaving at 7 bars/4 hours led to filling large with various types of highly crystalline hydration products, such as huge needles of CSH, very thin and stacked sheets of CASH, and a spherical zeolitic phase^44,77^. These deposits certainly contributed to shifting the textures/internal-surfaces of C-10B10S pastes from a wide to a mesoporous system^54^ as demonstrated previously (see Sect. 3.3); hence, the resistance to compression has improved from 61.5 to 72 MPa.

**Fig. 10:**
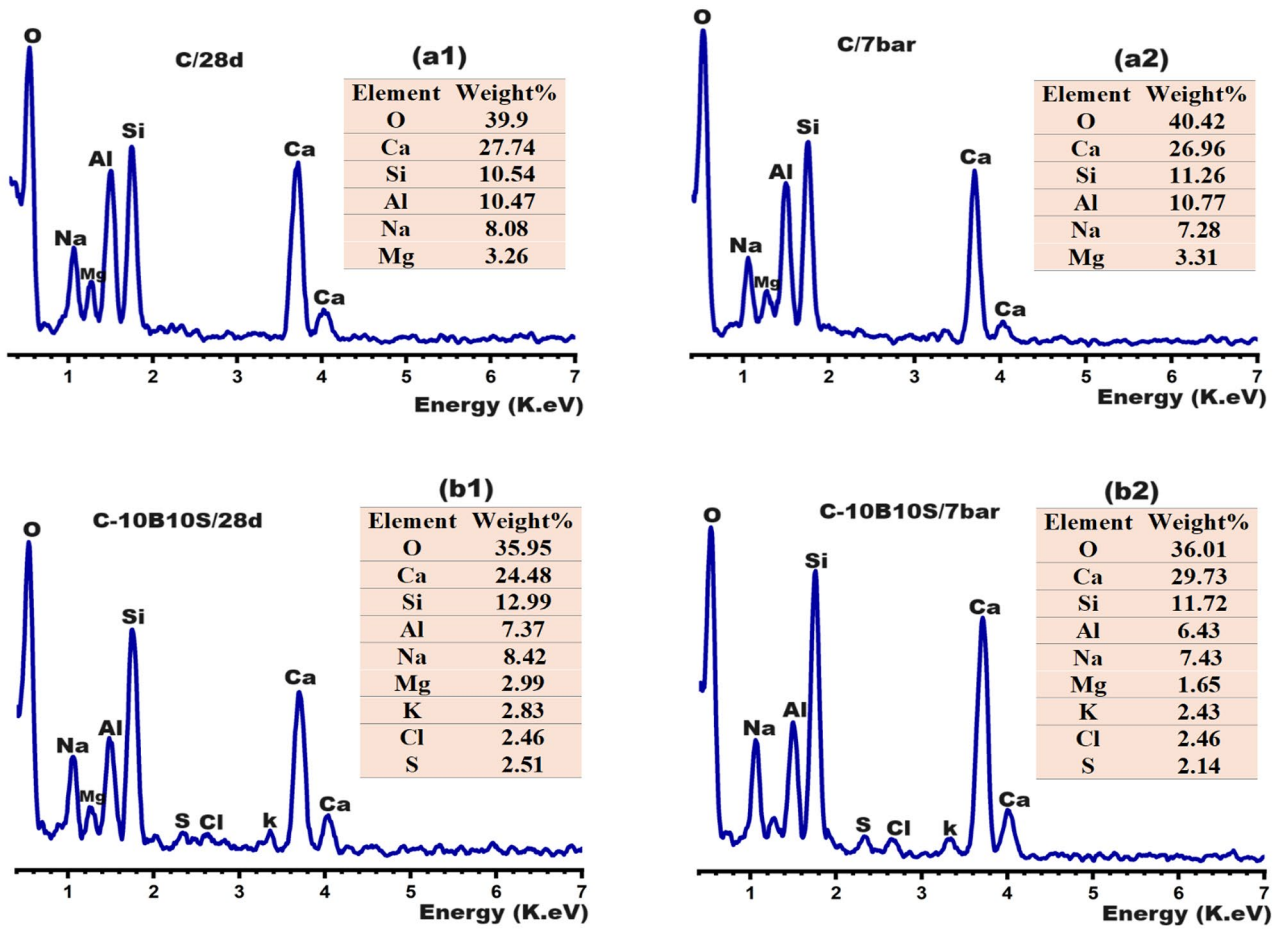
EDX-images for the selected composites at different curing conditions.

Figure 10 illustrates EDX images of C and C-10B10S pastes at different curing regimes. The elemental analysis confirms the existence of O, Si, Al, Ca, Mg, and Na as the main elements inside the structure of all pastes at normal and hydrothermal curing, while K, Cl, and S represent minor percentages inside the structure of C-10B10S, which contained 10% bypass. It is worth noting that bypass waste contained sylvite phase (KCl) and sulfate-containing phases (as mentioned in Fig. 1; Table 1). As clarified in Fig. 10 (a1, b1), Ca/Si ratios are 2.63 and 1.88 for C and C-10B10S pastes and Ca/Al ratios are 2.64 and 3.32 while Na/Al ratio are 0.77 and 1.14, respectively under normal curing. These ratios represent semi-quantitative analysis for the formed CSHs, CAH/CASHs and NASHs. The obtained data confirms that C-pastes possessed the highest amounts of CSHs while C-10B10S possessed the highest amounts of calcium aluminate-containing phases (CAH or CASH) and sodium alumino silicate hydrates^15^. Figure 10 (a2, b2) indicates that these ratios were changed under hydrothermal curing because of the recrystallization^53^ of most formed binding hydrates in the form of needle CSHs, tetragonal hydrogarnet, and hexagonal plates of CASH in addition to generating analcime which reinforced the compressive strength.

### Dye removal application

**Fig. 11:**
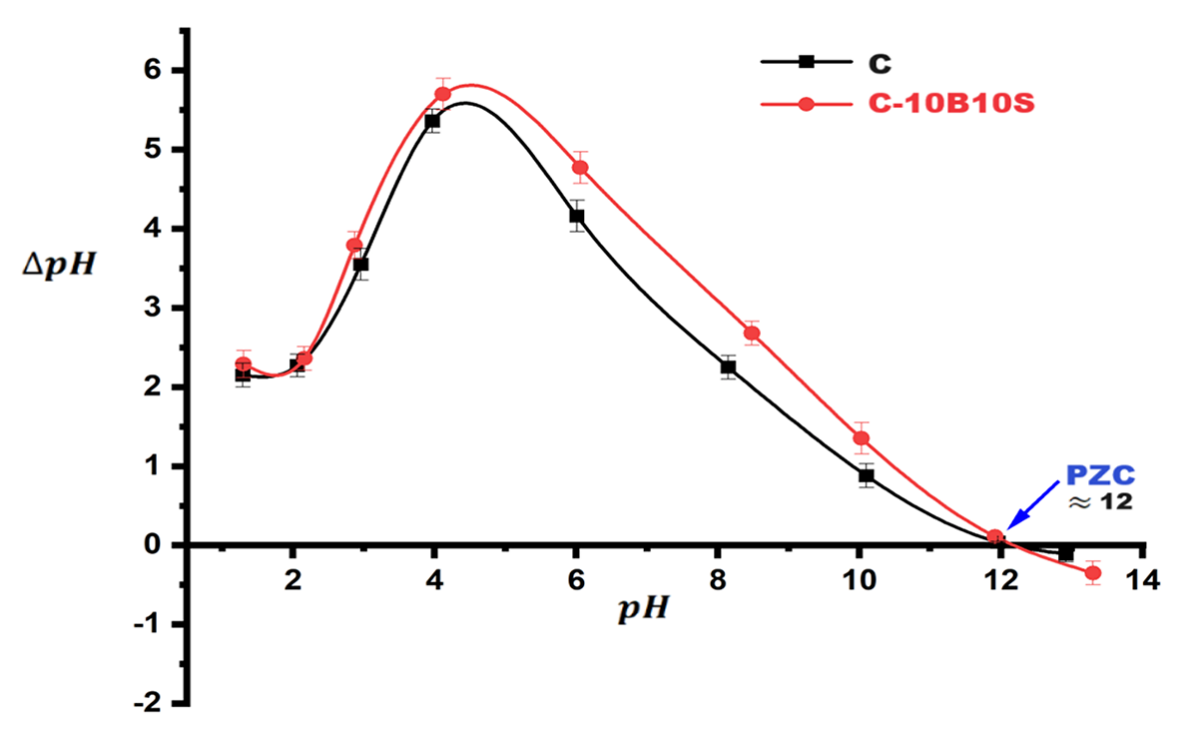
Point of zero charge (PZC) plot of autoclaved C and C-10B10S adsorbents.

**Fig. 12:**
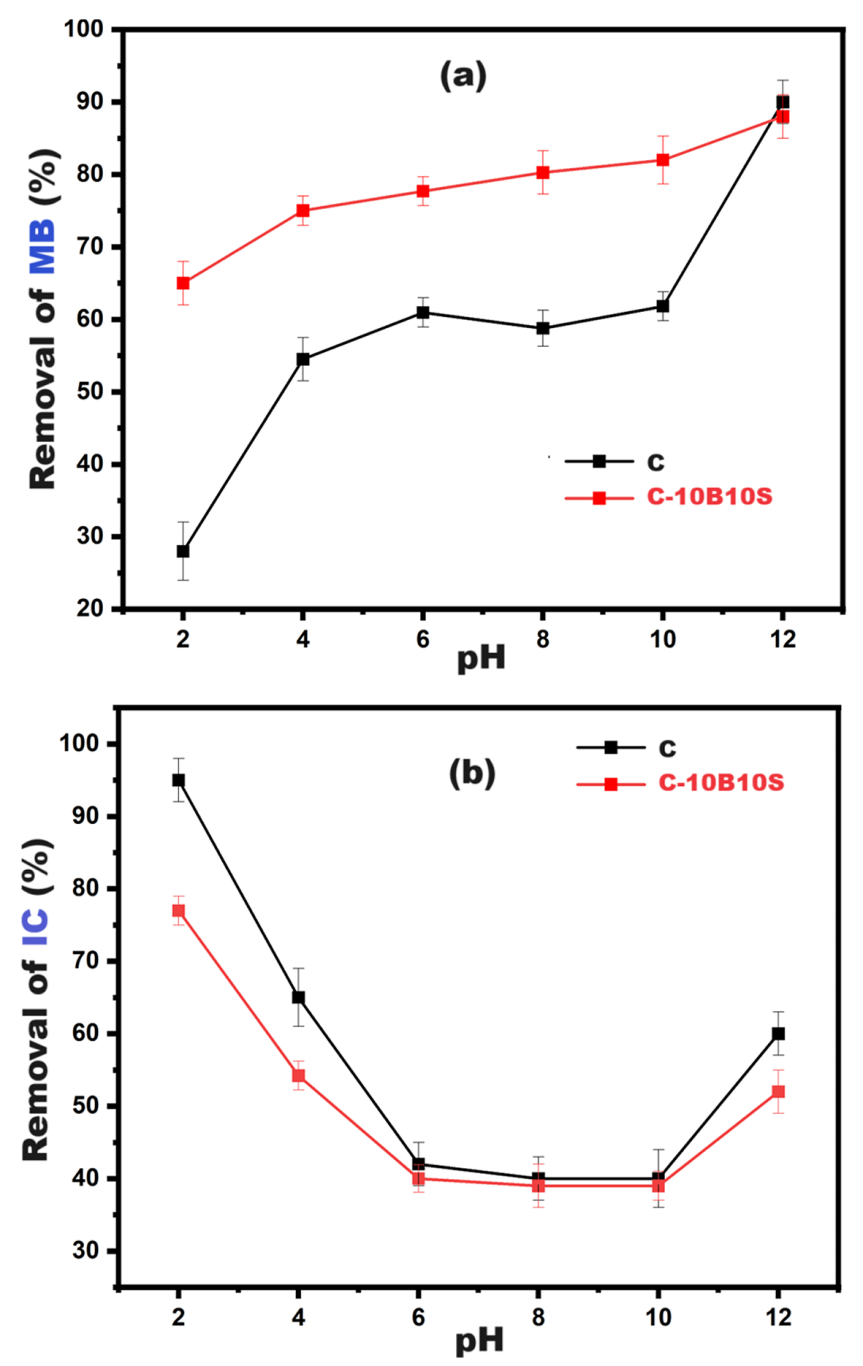
Effect of pH solution on **(a)** MB removal and **(b)** IC removal.

In this section, we discuss the suitability of using the prepared alkali-activated materials (C and C-10B10S) for special applications, for example, as adsorbents to remove anionic and cationic dyes. The hydrothermally cured powders at 7 bars/4hours were selected to conduct this section due to their high surface properties in terms of area and meso-porosity. Figure 11 confirms that the point of zero charge (PZC) for both adsorbents is $\:\approx\:$ 12. At this pH, the net charge on the surface of C and C-10B10S equals zero. Therefore, according to previous studies^78,79^, these adsorbents are expected to be more efficient in removing anionic dyes than cationic dyes. In this investigation, two different types of dyes were used, namely, methylene blue (MB) as a cationic dye and indigo carmine (IC) as an anionic dye. The selection of these dyes was based on their negative impacts on humans as well as the environment, as mentioned in the literature survey. It is worth noting that the electrical and porous nature of alkali-activated alumino-silicate materials is greatly affected by pH values. Therefore, the impact of the solution pH on MB and IC uptake by C and C-10B10S adsorbents was studied in the pH range 2–12, and the results are clarified in Fig. 12 (a, b). Figure 12-a illustrates the efficiency of adsorbents for removing the MB dye at pH = 2, 4, 6, 8, 10, and 12. A continuous increase was observed in removal% with increasing pH up to 12. As discussed previously (Sect. 3.3 and 3.5), the main hydration and activation products inside the structure of C and C-10B10S are the highly crystalline CSHs, CAH, CASH, and analcime. So, the main active sites that are responsible for dye removal are Si-O-Si, Si-O-Al, Si-OH, Al-OH, and Si-O^−^, in addition to the negative charge on the Al atom within zeolite networks^44,45^. Most of these sites are protonated (positively charged surfaces) at lower pH, so strong electrostatic repulsion takes place, which retards the removal of cationic dye (MB). At pH = 2, a minimum uptake was recorded due to the maximum protonation and repulsion. The deprotonation increases with increasing pH values up to 12, at which point the net charge on the adsorbent’s surface is $\:\approx\:$ zero. The maximum MB uptake was observed at pH = 12 due to (i) electrostatic attraction between Si-O sites and MB; (ii) electrostatic attraction between negatively charged Al sites and MB; (iii) H-bond between Si-OH and Al-OH and MB, (iv) dipole-dipole interaction between Si-O-Si and Si-O-Al and MB^80,81^. As indicated in Fig. 12-a, the removal efficiency of C-10B10S toward MB is relatively higher than the corresponding performance of C-adsorbent in the pH range of 2–10. This behavior is attributed to the hydration products, as the quantities in the case of C-10B10S are relatively lower than those of C (calculated from TGA results), so the degree of protonation and electrostatic repulsion in the case of C-10B10S are relatively lower than those of C, so the degree of protonation and electrostatic repulsion in the case of C-10B10S is relatively lower than that of C-adsorbent. Table 7 confirms that the zeta potential values of C-10B10S at pH 2 and 8 are + 4.30 and + 1.51 mV, respectively, while the control adsorbent (C) possesses + 7.68 and + 2.01 mV at the same pH values. Certainly, increasing the positive potential is evidence of an increase in the number of protons on the adsorbent’s surface, which increases the force of repulsion with the MB dye and thus becomes difficult to remove. A turning point was detected at pH = 12, where a maximum uptake was observed. At this point, the net charge on the surface of two adsorbents is near zero. It was found that the zeta potential values of C-10B10S and C are 0.00 and − 0.06 mV, respectively. Therefore, the main factor affecting the adsorption process at pH = 12 is the porous nature (meso-porosity) and not electrostatic forces^74^. The recorded removal percentages of MB by C-10B10S and C are 88 and 90%, respectively. As clarified in Table 4, the specific BET-surface area (S.A.) and total pore volume (Vt) in the case of C adsorbent are relatively higher than the corresponding values of C-10B10S.

Figure 12-b shows the efficiency of adsorbents for removing the IC dye. A continuous decrease was observed in removal% with an increasing pH up to 10, followed by a slight increase at pH 10–12. At pH = 2, a maximum IC uptake is recorded due to the maximum protonation, accompanied by maximum electrostatic attraction with the anionic dye. Increasing the pH value up to 10 causes a strong decay in the number of protons on the surface of the selected adsorbents; hence, the removal efficacy decreases. The slight increase in removal percentage at pH 12 is attributed to the meso-porosity nature of the two adsorbents. The situation was completely reversed in the case of removing IC dye, as the control sample (C) became more efficient compared to the modified sample (C-10B10S). This is attributed to the high surface characterization and high electrostatic nature of the control sample (see Table 7), which contributed strongly to the removal of anionic dyes.

**Table 7:** Zeta potential and textural parameters for C and C-10B10S adsorbents at different pH values.

Selected paste	pH	Zeta potential (mV)	BET-S.A. (cm^2^/g)	Vt (cm^3^/g)
C	2	+ 7.68	42,534	0.1356
	8	+ 2.01	35,313	0.1317
	12	-0.06	33,784	0.1300
C-10B10S	2	+ 4.30	34,218	0.1059
	8	+ 1.51	14,908	0.0683
	12	0.00	13,395	0.0579

## Conclusion

In this study, five distinct eco-friendly pastes (alkali-activated cement) based on slag, bypass (B), and silica fume (S) have been fabricated. The mechanical behavior (represented by compression resistance) and efficiency of the prepared pastes for removing cationic and anionic dyes from aqueous mediums were studied.

### The outcomes of this study can be summarized as follows

- The alkali-activated cement pastes subjected to normal conditions (~ 98% humidity at room temperature) indicated a continuous strength development up to the final curing age (90 days). This strength development is assigned to the formation of phases like (CSHs), (CASHs), (CAHs), (NASHs), and (Ht), as revealed by XRD and TGA/DTG techniques.
- The hydrothermal curing (steam curing in an autoclave at 7 and 10 bar) of the prepared green pastes for 240 min causes notable improvements in their mechanical characteristics compared with the achieved strength at 28 days under normal curing conditions. As the curing pressure increases, the quantities of the force-giving phases (CSHs, CASHs, CAHs, NASHs, and Ht) increase, so the compressive strength values increase.
- From the economic point of view, the optimum hydrothermal curing condition according to the obtained strength values was (7 bar/178^o^C) for 240 min.
- The optimum composite design that showed the best mechanical behavior under both curing regimes (normal and hydrothermal) is C-10B10S.
- The prepared alkali-activated cement pastes (C and C-10B10S) showed good efficiency for wastewater treatment. They can be used as efficient adsorbents for both cationic and anionic dyes.
- The efficiency of alkali-activated adsorbents for removing the MB (as cationic dye) increases with the pH value reaching its maximum at pH = 12. While it decreases as pH increases in the case of IC as an anionic dye.

## Data Availability

The datasets used and/or analysed during the current study available from the corresponding author on reasonable request.
